# Simple Preparation of Conductive Hydrogels Based on Precipitation Method for Flexible Wearable Devices

**DOI:** 10.3390/s25196032

**Published:** 2025-10-01

**Authors:** Bolan Wu, Jiahao Liu, Zunhui Zhao, Na Li, Bo Liu, Hangyu Zhang

**Affiliations:** School of Biomedical Engineering, Liaoning Key Lab of Integrated Circuit and Biomedical Electronic System, Faculty of Medicine, Dalian University of Technology, Dalian 116024, China; 2251740842@mail.dlut.edu.cn (B.W.); ljh2356486741@163.com (J.L.); zhaozh_dut@163.com (Z.Z.); lina316@dlut.edu.cn (N.L.); lbo@dlut.edu.cn (B.L.)

**Keywords:** conductive hydrogels, precipitation, facile preparation, strain sensor, bioelectrodes

## Abstract

**Highlights:**

**What are the main findings?**

- A novel precipitation method via solution blending and centrifugation was developed for the facile preparation of conductive polymer composite hydrogels (e.g., PEDOT/PAA/PVA).
- The prepared hydrogels exhibit excellent electrical (conductivity: 4.065 S/m) and mechanical (Young’s modulus: 311.6 kPa) properties, and perform well as strain sensors (sensitivity: 1.86; response time: 400 ms) and bioelectrodes (lower contact impedance than commercial electrodes, and showed no signs of skin irritation under tested conditions).
- The method shows universal applicability for different conductive polymers (e.g., PANI, PPy) and hydrogel substrates (e.g., PVA, PAAm).

**What is the implication of the main finding?**

- It provides a universal, simple, and low-cost strategy for rapid synthesis of customizable conductive hydrogels, overcoming limitations of traditional complex methods.

**Abstract:**

Conductive polymer hydrogels have attracted extensive attention in wearable devices, soft machinery, and energy storage due to their excellent mechanical and conductive properties. However, their preparation is often complex, expensive, and time-consuming. Herein, we report a facile precipitation method to prepare conductive polymer composite hydrogels composed of poly(acrylic acid) (PAA), poly(vinyl alcohol) (PVA), and poly(3,4-ethylenedioxythiophene) (PEDOT) via straightforward solution blending and centrifugation. During the preparation, PEDOT, grown along the PAA template, is uniformly dispersed in the hydrogel matrix. After shaping and rinsing, the PEDOT/PAA/PVA hydrogel shows good mechanical and electrical properties, with a conductivity of 4.065 S/m and a Young’s modulus of 311.6 kPa. As a strain sensor, it has a sensitivity of 1.86 within 0–100% strain and a response time of 400 ms. As a bioelectrode, it exhibits lower contact impedance than commercially available electrodes and showed no signs of skin irritation in the test. The method’s versatility is confirmed by the observation of similar performance of hydrogels with different compositions (e.g., polyaniline (PANI)/PAA/PVA). These results demonstrate the broad applicability of the method.

## 1. Introduction

With rising health awareness, the traditional rigid medical equipment is no longer adequate to address the diverse and evolving demands of people. Wearable flexible electronic devices are gradually being more widely concerned [1,2]. These newly developed flexible wearable electronics are like a bridge between humans and machines, and they can be applied to the skin surface as sensors or patch electrodes to monitor physiological parameters and treatment effects of patients, providing an important auxiliary means for medical diagnosis and treatment [3,4,5].

It is noteworthy that conductive polymer composite hydrogels show great advantages in the manufacture of bioflexible electronic devices because of their excellent conductivity and biocompatibility [6,7,8]. The formation of soft conductive hydrogels is achieved through the incorporation of conductive fillers or conductive polymers within an insulating hydrogel substrate, which can be facilitated by the introduction of multivalent metal ions or through post-processing of the resultant polymers [9,10,11,12,13,14,15,16,17,18,19,20,21,22]. These conductive hydrogels are tunable and designable, allowing their properties (e.g., mechanical properties) to be adapted to the environment in which they are to be used through the adjustment of formulations and the choice of materials [20,21,23,24]. In a notable study, Wei et al. constructed a conductive polymer composite hydrogel by in situ polymerization of aniline monomers in polyvinyl alcohol (PVA), followed by cross-linking of PVA by glutaraldehyde (GA) as a cross-linking agent to act as a wearable sensor. This sensor enabled the detection of different movements of the human body and even the differentiation of speech content [20]. The distinctive characteristics of conductive hydrogels make them a promising class of materials for a wide range of applications.

At present, the preparation of conductive polymer hydrogels can be achieved through a variety of methodologies, including electrochemical preparation, soaking preparation, dispersion preparation, and solution processing. Electrochemical polymerization involves the infiltration of conductive polymer monomers into a system of hydrophilic hydrogels through the use of electrodes. It was reported that a sulfhydryl self-assembly layer was grafted onto the surface of titanium using gelatin methacrylate, and subsequently, a conductive polypyrrole layer was introduced through the electrochemical method. The conductive hydrogel coating prepared by this method has been shown to exhibit excellent electrochemical properties and biocompatibility [25,26,27,28]. Immersion preparation method is one of the most popular methods for preparing conductive polymer hydrogels. Conductive polymer monomers and oxidants are mixed together and infiltrated into the prepared hydrogel network by means of immersion. Wang et al. introduced aniline into conductive polymer hydrogel prepared in PAA hydrogel system by immersion method to prepare strain sensor for detecting human behavior [21,29]. The dispersion preparation method involves the dispersion of a conductive polymer, which was prepared in advance, in a hydrogel precursor solution by means of ultrasound, followed by the initiation of hydrogel network polymerization by heat curing to prepare the conductive polymer composite hydrogel [30]. The solution processing method is a new synthesis method. Firstly, the hydrogel polymer, the conductive polymer monomer and the oxidizer are mixed. Thereafter, the conductive polymer grows along the long chain of the hydrogel polymer. Subsequently, hydrogel polymer chains are crosslinked to synthesize the conductive polymer composite hydrogel by post-crosslinking [30].

The fundamental principle underlying these methods lies in the sequence of conductive polymer synthesis relative to hydrogel network formation. Conductive polymers are either grown along pre-existing hydrogel templates or dispersed within the precursor solution. However, each method presents certain limitations. For instance, the soaking process is often time-consuming and may lead to hydrogel swelling, while dispersion methods can result in inhomogeneous distribution of the conductive polymer. Increasing the concentration of conductive polymers within the hydrogel remains challenging. These issues complicate the fabrication process and may restrict the practical applications of such hydrogels. Thus, developing a simple, low-cost, and efficient preparation strategy that balances mechanical and electrical properties continues to be a significant challenge [31,32,33,34].

In this study, we reported a low-cost and simple preparation method of conductive polymer composite hydrogels. The conductive polymer composite hydrogels, based on polyacrylic acid (PAA) and polyvinyl alcohol (PVA), were successfully prepared by means of simple solution blending and deposition. The conductive polymer composite hydrogel was formed by growing the conductive polymer along the long chain of the hydrogel polymer and precipitating and concentrating together with the hydrogel polymer. This method of preparation was designed to avert the occurrence of conductive polymer aggregation, which could result from an excess of conductive polymer monomer during the polymerization process. Compared to conventional multi-step methods such as soaking or dispersion preparation, which often require hours or specialized equipment, our precipitation strategy offers a remarkably facile and rapid route to functional conductive hydrogels. Additionally, it ensured the incorporation of conductive polymer into the concentrated hydrogel. The prepared hydrogels were successfully packaged as strain sensors, which could be used to detect movements. The hydrogels could also be used as bioelectrodes to detect electrocardiogram (ECG) or electroencephalogram (EEG) signals. This method was proved to be universally applicable and could be employed in a variety of conductive polymer materials and hydrogel substrates.

## 2. Materials and Methods

### 2.1. Materials

Sodium polyacrylate (PAAS, Mw 3000–7000 kDa) was purchased from Shanghai Xianding Biotechnology Co., Ltd. (Shanghai, China). Polyvinyl alcohol (PVA-224), 3,4-ethylenedioxythiophene (EDOT, 99%), aniline (≥99.5%), glutaraldehyde (GA, 50%), dopamine hydrochloride (98%), ammonium persulfate (APS, 98.5%), ferric trichloride hexahydrate (99%), pyrrole (Py, 99%), polyacrylamide (PAAm, Mw 7000 kDa) were all purchased from Shanghai Macklin Biochemical Technology Co., Ltd. (Shanghai, China). Sodium hydroxide (AR) was purchased from Sinopharm Chemical Reagent Co., Ltd. (Shanghai, China). Urea (99%, note: original text incorrectly wrote “urea nitrogen”) was purchased from Shanghai Tengzhun Biotechnology Co., Ltd. (Shanghai, China). Citric acid (AR, ≥99.5%) was purchased from Shanghai Boer Chemical Reagent Co., Ltd. (Shanghai, China). Anhydrous sodium citrate (≥99%) was purchased from Shanghai Dibo Biotechnology Co., Ltd. (Shanghai, China). Sodium chloride (99.8%) was purchased from Shanghai Aladdin Biochemical Technology Co. Ltd. (Shanghai, China).

### 2.2. Preparation of PEDOT/PAA/PVA Hydrogels

A FeCl_3_-HCl solution was prepared by mixing 20 mL of pH 0 HCl solution with 1 mL of 10% (*w*/*w*) FeCl_3_ solution. After thorough mixing of the FeCl_3_-HCl solution with 1% (*w*/*v*) PAA solution, 10% (*w*/*w*) APS and EDOT monomer were added. The mixture was shaken at room temperature for 24 h using a shaking machine to obtain PEDOT/PAA solution. 6 mL of PEDOT/PAA solution was mixed with 2.3 mL of 1% (*w*/*v*) PVA solution, thoroughly blended, and centrifuged for 3 min. The precipitate was collected as PEDOT/PAA/PVA hydrogel. After absorbing surface water with non-woven cloth, the hydrogel was placed into a glass fixture with a 300 μm spacing and oven-dried at 60 °C for 15 min for dehydration and solidification. Subsequently, 8 μL of an aqueous glutaraldehyde (GA) solution (5% *w*/*w*) was evenly drop-cast onto the surface of the hydrogel to facilitate chemical cross-linking, which was allowed to proceed at room temperature for 10 min. After the reaction was complete, the hydrogel was retrieved. A cleaning solution was prepared by dissolving 50.4 g FeCl_3_, 36 g NaCl, 360 g urea, 17.5 g citric acid (CA), and 100.3 g sodium citratein 2.4 L deionized (DI) water with thorough stirring. The PEDOT/PAA/PVA hydrogel cross-linked with glutaraldehyde was repeatedly immersed in 50 mL of this cleaning solution.

### 2.3. Preparation of PAA/PVA Hydrogels

6 mL of FeCl_3_-HCl solution was mixed with 2.3 mL of PAA solution, thoroughly agitated, and centrifuged for 3 min. The precipitate was collected as PAA/PVA hydrogel, which was then shaped, soaked, and cleaned to obtain the final product.

### 2.4. Material Characterization

Fourier transform infrared (FT-IR) spectroscopy: Performed using a Thermo Scientific Nicolet IS50 (Waltham, MA, USA) in the wavelength range of 4000~400 cm^−1^.

X-ray photoelectron spectroscopy (XPS): Acquired using a Thermo Scientific ESCALAB Xi+ instrument (Waltham, MA, USA) with scanning in the 1350~0 eV interval for compositional analysis of N, C, and S elements.

Samples (PEDOT/PAA/PVA and PAA/PVA hydrogels) were lyophilized using a BIOCOOL vacuum freeze dryer FD-1A-80 (BIOCOOL, Beijing, China) before characterization.

### 2.5. Conductivity Test

PEDOT/PAA solutions were prepared as per Table 1. Hydrogels were cut into 10 mm × 10 mm × 0.3 mm squares, sandwiched between two electrode pads, and subjected to alternating current (AC) impedance testing using a CHI660E electrochemical workstation (Shanghai Chenhua Co., Ltd., Shanghai, China) in the 10^−1^ to 10^4^ Hz frequency range.

Sheet resistance was measured using a four-probe tester (RTS-9, 4Probes Tech Ltd., Guangzhou, China). Conductivity (κ) was calculated as:

κ = 1/(d × R□) (1)

where R□ is the sheet resistance (Ω/□) and d is the hydrogel thickness (m).

### 2.6. Skin Contact Impedance Measurement of Hydrogels

Three 10 mm × 10 mm × 0.3 mm PEDOT/PAA/PVA hydrogels were applied to the inner side of the forearm at 10 cm intervals, connected to the working, reference, and counter electrodes of an electrochemical workstation, respectively, and subjected to AC impedance testing in the 10^−1^–10^4^ Hz range.

### 2.7. Mechanical Property Test

Hydrogels were trimmed into 30 mm × 10 mm × 0.3 mm strips, fixed to a universal testing machine ZHIQU ZQ-990B (Dongguan, China) clamps, and subjected to uniaxial cyclic tensile tests at 100 mm/min within 0–20%, 0–40%, 0–60%, 0–80%, and 0–100% strain.

Stress (σ): σ = F/S (2)

where F is the load (N) and S is the cross-sectional area (mm^2^).

Strain (ε): ε = (L − L_0_)/L_0_ × 100% (3)

where L is the elongated length (mm) and L_0_ is the initial length (mm).

Young’s modulus (E): E = σ/ε (4)

Calculated using two points in the linear region of the stress–strain curve.

### 2.8. Strain Sensor Performance Test

A 30 mm × 10 mm × 0.3 mm hydrogel strip was fixed to a universal testing machine, with copper tape at both ends connected to a portable precision resistance/capacitance measuring device (TruEbox 01RC, LinkZill, Hangzhou, China) at 250 kHz.

The gauge factor (GF) can be expressed as the rate of change in resistance (ΔR/R_0_) in the range of 0 to 200% for samples tested at 5% tensile deformation on each occasion.

Gauge factor (GF) was calculated as:

GF = (ΔR/R_0_)/(ΔL/L_0_) (5)

ΔR = |R − R_0_| (6)

ΔL = |L − L_0_| (7)

R is real-time resistance, R_0_ is initial resistance, L is real-time length, and L_0_ is initial length. Tests included variable amplitude stretching (5%, 15% strain at 1 Hz), variable frequency stretching (10% strain at 0.25 Hz and 0.5 Hz), and 100 cyclic loading tests (10% strain at 1 Hz).

### 2.9. Bioelectrode Performance Test

Skin irritation test: Hydrogel electrodes and commercial ECG electrodes were attached to the inner side of the forearm for 4 h, then removed to check for redness, swelling, or allergies. This test was conducted to preliminarily assess the short-term skin compatibility of the hydrogel electrodes.

ECG signal acquisition: Hydrogel electrodes (0.3 mm thick, 10 mm diameter) and commercially available ECG electrodes were attached to the inner side of the forearm using PU tape and connected to an ECG signal collector. Signals were recorded synchronously. The cosine similarity of signals was calculated to verify reliability:

(8) $$cos(\theta )=\sum_{i=1}^{n}A_{i}\times B_{i}/\left(\sqrt{\sum_{i=1}^{n}{(A}_{i}{)}^{2}}\times \sqrt{\sum_{i=1}^{n}{(A}_{i}{)}^{2}}\right)$$

where A_i_ and B_i_ are voltage values of commercial and hydrogel electrodes at the i-th sampling point, respectively.

EEG signal acquisition: A hydrogel electrode (1 mm thick, 10 mm diameter) was placed at the right prefrontal (Fp2), and the EEG paste was injected into the left prefrontal (Fp1). EEG signals were recorded synchronously, and cosine similarity was calculated. The impedances of the electrodes were recorded hourly to monitor the long-term performance.

### 2.10. Universality of the Preparation Methods

PANI/PAA/PVA and PPy/PAA/PVA hydrogels: Conductive polymer monomers (aniline or pyrrole), PAA, HCl, FeCl_3_, and APS were mixed, stirred at 4 °C until reaction completion to obtain PANI/PAA or PPy/PAA solutions, which were then mixed with PVA solution. Precipitates were collected by centrifugation.

PEDOT/PAA/PAAm hydrogels: 6 mL PEDOT/PAA solution was mixed with 2.3 mL 1% (*w*/*v*) PAAm solution, centrifuged, and the precipitate was collected.

PDA/PVA/PAA hydrogels: A 0.5% (*w*/*v*) PVA solution (pH 11) with 20 mg dopamine hydrochloride was shaken at room temperature for 12 h to obtain PDA/PVA solution. 2.5 mL 1% (*w*/*v*) PAA solution was mixed with 125 μL pH 0 HCl and 1 mL FeCl_3_ solution to form FeCl_3_-PAA solution. The two solutions were mixed, and the precipitate was collected by centrifugation.

PANI/PAA/PVA hydrogels were tested as ECG electrodes similarly to PEDOT/PAA/PVA, with cosine similarity calculated for signals.

## 3. Results and Discussion

### 3.1. Preparation of PEDOT/PAA/PVA Hydrogels

**Figure 1:**
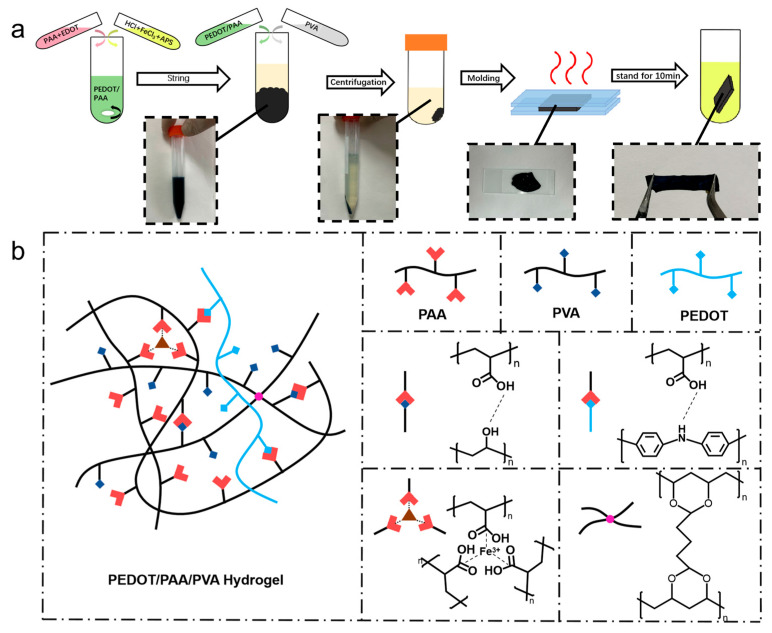
Schematic diagram of (**a**) PEDOT/PAA/PVA hydrogel preparation process and (**b**) iteraction of components of PEDOT/PAA/PVA hydrogels.

The precipitation method of preparing conductive polymer hydrogels was illustrated in Figure 1a and PEDOT was utilized first as a model conductive polymer for this universal method. Initially, EDOT and PAA were combined at room temperature with continuous stirring initiated subsequent to the addition of Ammonium Persulfate (APS), HCl and FeCl_3_. EDOT was then grown into PEDOT/PAA with good solubility using PAA as the template and the dopant under the oxidation of FeCl_3_ and APS. The incorporation of HCl provided an acidic condition for the polymerization of EDOT, lowered the viscosity of the PAA solution, and prevented PAA from precipitation due to the liganding effect of Fe^3+^. Subsequently, the prepared PEDOT/PAA solution was mixed with the PVA solution, leading to the interaction between PEDOT/PAA and PVA through hydrogen bonding and their entanglement, and ultimately yielding a blue-black, fluffy precipitate. On the other hand, the solution itself became yellowish transparent, indicating that the majority of the PEDOT precipitated together with the hydrogel network, rather than remaining in the solution. The precipitate was collected by centrifugation and allowed to dehydrate further, resulting in the formation of a PEDOT/PAA/PVA hydrogel. The hydrogel underwent a heating and dehydration process, with the hydrogel sandwiched between two glass slides separated by a distance of 300 μm. The dehydrated hydrogel was further treated with GA for a quick crosslinking followed by the immersion in cleaning solution, resulting in a transition from plasticity to elasticity. Figure 1b illustrates the proposed interactions between the components of the hydrogel. This uniform network is achieved because PEDOT grows in situ along the molecularly dispersed PAA chains. This result aligns with accounts found in most published studies [35,36,37]. Subsequently, the strong hydrogen bonding between PAA and PVA, synergizing with the Fe^3+^ ionic cross-linking, ensures the integrated co-precipitation and homogeneous distribution of the conductive polymer within the resulting hydrogel matrix. The ternary network structure and intricate interactions endowed the hydrogel with excellent structural stability.

**Figure 2:**
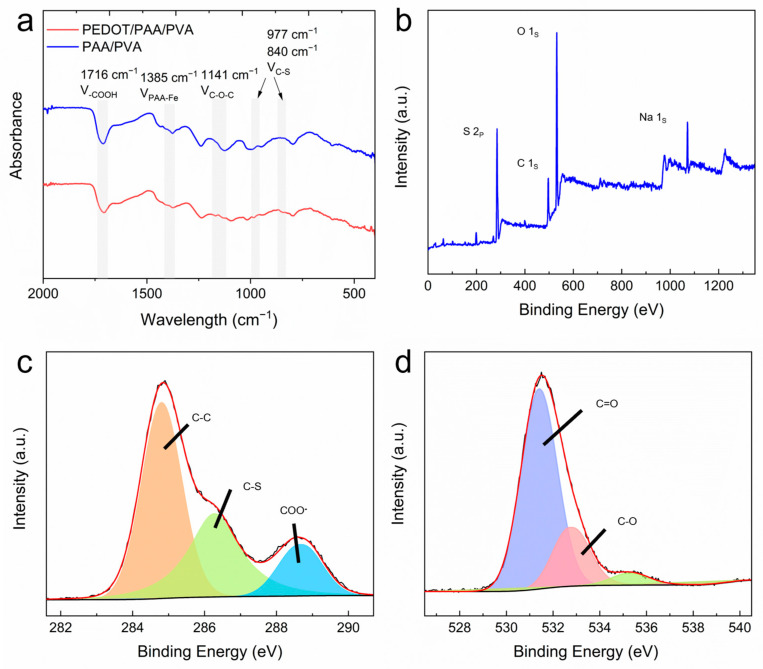
(**a**) FTIR spectra of dried hydrogels. XPS survey spectrum (**b**) and high-resolution XPS spectra of C 1s (**c**) and O 1s (**d**) of PEDOT/PAA/PVA.

Fourier transform infrared (FTIR) and X-ray photoelectron spectroscopy (XPS) spectra of PEDOT/PAA/PVA hydrogels and PAA/PVA hydrogels after lyophilization were collected to gain insight into the components present in the hydrogel and the interactions between them. As illustrated in Figure 2a, distinctive absorption peaks were observed at 1716 cm^−1^ and 1385 cm^−1^ in the FTIR spectra of both PEDOT/PAA/PVA and PAA/PVA hydrogels, attributed to -COOH and PAA-Fe, respectively, supporting that PAA was physically crosslinked by Fe^3+^. The characteristic peaks of C-O-C at 1141 cm^−1^ and C-S at 977 cm^−1^ and 840 cm^−1^ in the spectrum of PEDOT/PAA/PVA hydrogels indicated the formation of PEDOT [36,38,39,40,41,42]. XPS analysis (Figure 2b–d) revealed the presence of S 2p, C 1s, O 1s, and Na 1s peaks in the hydrogel. The XPS spectra of C 1s were convolved to three peaks that could correspond to 285.0 eV (C-C), 286.4 eV (C-S), and 288.7 eV (-COO-), respectively. Furthermore, the XPS spectra of O 1s were convolved to peaks that could correspond to 531.5 eV (C=O) and 533 eV (-OH), indicating the presence of PEDOT, PAA, and PVA [43]. The predominant source of sodium was inferred to be NaCl in the cleaning solution, while the source of sulfur was identified as PEDOT in the hydrogel.

### 3.2. The Characteristics of PEDOT/PAA/PVA Hydrogels

**Figure 3:**
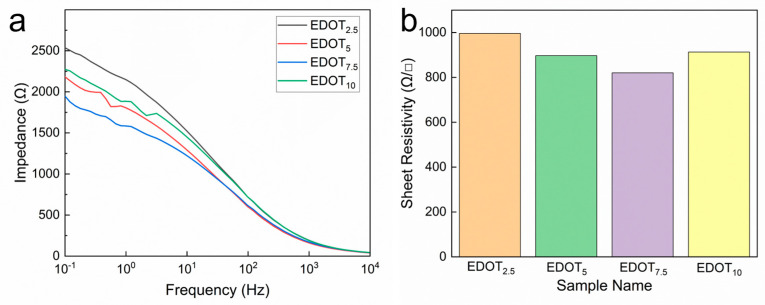
Impedance spectra (**a**) and sheet resistance (**b**) of PEDOT/PAA/PVA hydrogels with different EDOT dosage.

Electrical properties are an important index for the evaluation of bioelectrodes. The impedance of PEDOT/PAA/PVA hydrogels was tested by sandwiching the hydrogel sheets with varying EDOT dosages between copper tapes within the frequency range of 10^−1^ Hz to 10^4^ Hz, utilizing an electrochemical workstation. As illustrated in Figure 3a, the impedance of PEDOT/PAA/PVA hydrogels decreased as the amount of EDOT fed increased, until it reached 7.5 μL (EDOT7.5). However, when the EDOT dosage further increased to 10 μL, the impedance increased instead, which is speculated to be resulted from the aggregation of PEDOT. Furthermore, to assess the electrical property of PEDOT/PAA/PVA hydrogels, the sheet resistance was measured using the four-probe method. Similarly, PEDOT/PAA/PVA hydrogels exhibited the lowest sheet resistance value of 820 Ω/□ when the EDOT dosage was 7.5 μL and the conductivity is calculated to be 4.065 S/m (Figure 3b). Beyond this point, the impedance increased, likely due to the aggregation of PEDOT chains at higher concentrations, which can hinder charge transport by creating discontinuous conductive domains, a common challenge in conductive polymer composites [44,45]. Accordingly, the PEDOT/PAA/PVA hydrogel with EDOT dosage of 7.5 μL was selected for all subsequent investigation.

The conductive hydrogel material is anticipated to demonstrate resilience in the face of repeated loading cycles to cope with a variety of application scenarios. In order to assess the mechanical properties and stability of the hydrogel, it was affixed to the tensile tester fixture for uninterrupted stretching-unloading experiments. The amplitude of stretching was set at 20%, 40%, 60%, 80%, and 100%, respectively. The continuous cyclic tensile loading-unloading curves of PEDOT/PAA/PVA hydrogels at different strains demonstrate that the mechanical properties of the hydrogels within this interval exhibit good linearity and reproducibility. Furthermore, the hydrogels can be rapidly restored to their original state without any damage (Figure S1). The Young’s modulus of the hydrogel was determined to be 311.6 kPa.

It should be noted that the concentration of FeCl_3_ was found to be critical for effective precipitation and initial gel formation. Although not the main focus of this study, it was qualitatively observed that the mechanical properties of the resulting hydrogel were modulated by altering the FeCl_3_ concentration, as this parameter controls the physical cross-linking density of PAA. These observations underscore the role of Fe^3+^ in the network formation.

The PEDOT/PAA/PVA hydrogel, with a Young’s modulus of approximately 311.6 kPa and a conductivity of 4.065 S/m, performs competitively against recently reported conductive hydrogels for wearable sensing [35,46,47]. This balanced performance was achieved through a simple and rapid precipitation method, contrasting with the more complex synthesis routes typically required for such materials.

### 3.3. The Application of PEDOT/PAA/PVA Hydrogels

**Figure 4:**
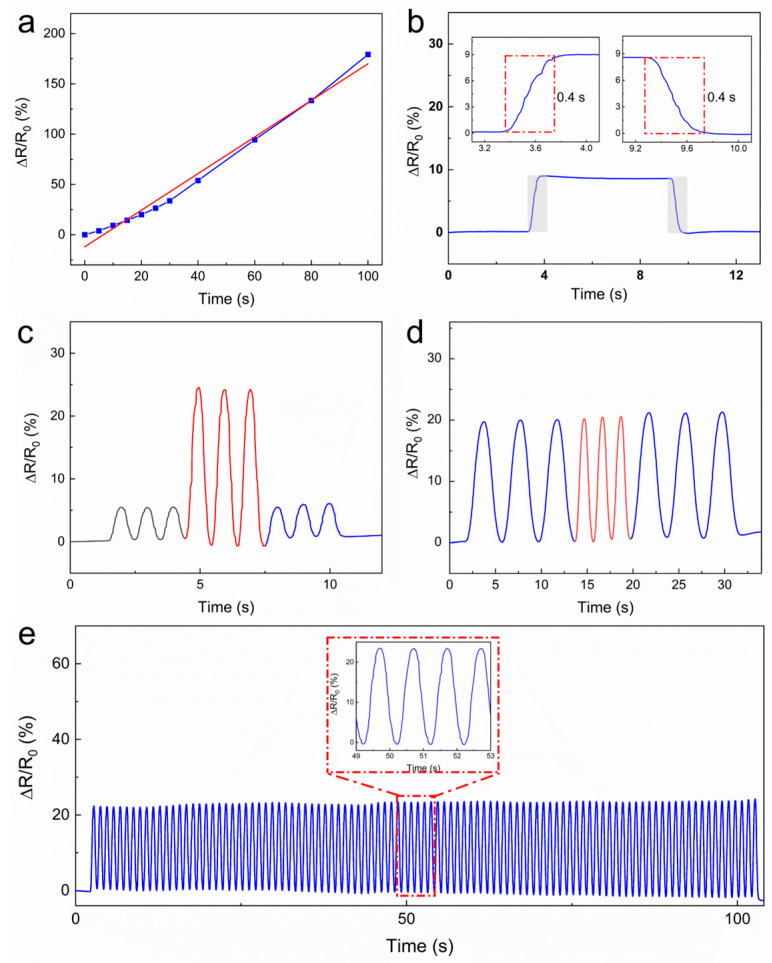
The performance of the PEDOT/PAA/PVA hydrogel as a strain sensor. (**a**) The electrical resistance and strain curve. The original data is illustrated by a blue line, while the linear-fitted is shown as a red dashed line. (**b**) The response and recovery times of the hydrogel. (**c**) The real-time response curve measured at 5% and 15% strains. (**d**) The real-time response curve measured at 0.25 and 0.5 Hz. (**e**) The cycling durability test of the hydrogel strain sensor.

Due to its excellent resistance to pressure, the hydrogel has been selected as a candidate for the fabrication of sensors. A piece of PEDOT/PAA/PVA hydrogel with dimensions of 30 mm in length, 10 mm in width, and 0.3 mm in thickness was extracted and affixed with copper tape at both extremities as electrodes. It was then encased in PU tape, thus creating a rudimentary sensor. The sensors were secured on a horizontal tensile stage and subjected to examination of their intrinsic electrical characteristics. Figure 4a demonstrated the resistance alteration rate of the hydrogel in the range of 0–100% strain corresponding to varying strains of the hydrogel. The hydrogel exhibited a sensitivity of 1.82 within this interval, which encompasses a broad detection range, high sensitivity, and robust stability. The linear response within the 0–100% strain range defines the effective sensing window for this sensor, which amply covers the requirements for detecting most human physiological activities and joint movements.

As illustrated in Figure 4b, the response time and relaxation time of the hydrogel were both 400 ms. The uniform response speed is further accompanied by consistent resistance after relaxation, suggesting a stable electrical property. As illustrated in Figure 4c,d, the hydrogel sensor exhibited stable and rapid responsiveness to cycle stretching at 5% and 15% strains with a frequency of 1 Hz, as well as to cycle stretching at a 10% strain with variable frequencies of 0.25 Hz and 0.5 Hz. Furthermore, the sensor exhibited consistent and effective response to cyclic tensile strain throughout 100 cycles of stretching at 10% strain (Figure 4e) with minimal signal degradation. The stability of over 100 cycles demonstrates promising durability for typical wearable applications. Investigation into ultra-long-term reliability (e.g., >1000 cycles) will be an important aspect of future work aimed at commercialization.

**Figure 5:**
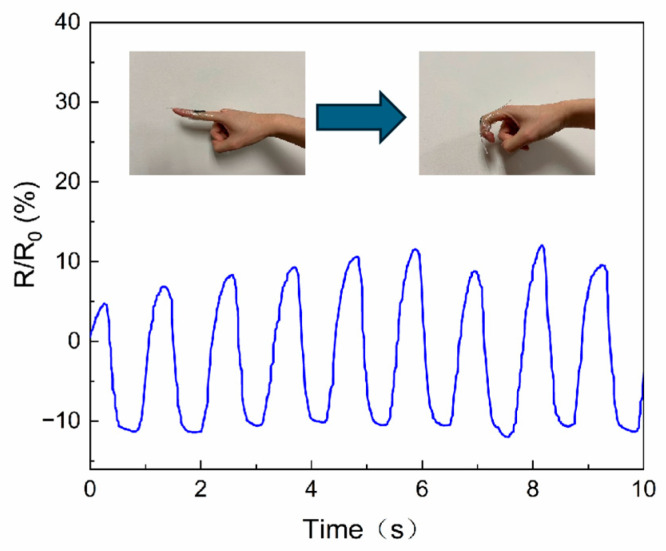
Finger joint motion measured by PEDOT/PAA/PVA hydrogel.

The hydrogels were then affixed to the joints of the right forefinger using PU tape encapsulation, and the resistance were altered corresponding to the movement of the finger in a consistent manner (Figure 5). The resistance change curve displayed stability and smoothness, and the absence of discernible drift phenomena.

**Figure 6:**
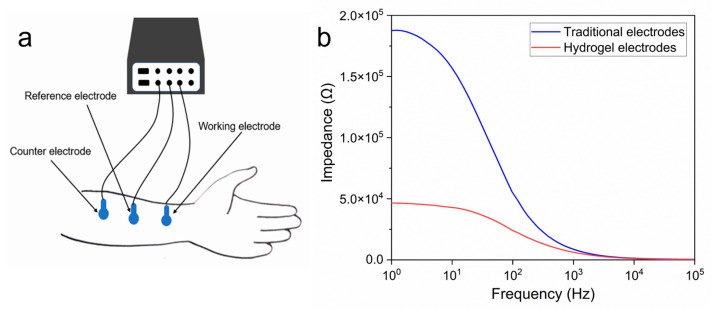
(**a**) Measurement of skin contact impedance; (**b**) Comparison of contact impedance between the PEDOT/PAA/PVA hydrogel electrode and commercial ECG electrodes.

PEDOT/PAA/PVA hydrogels were assessed for their capacity to function as bioelectrodes. The electrode-skin contact impedance was tested within the 10^0^–10^5^ Hz range (Figure 6a). The results demonstrated that the contact impedance of the hydrogel electrodes was markedly lower than that of the commercial electrodes (Figure 6b). This discrepancy could be attributed to two factors: the exceptional conductivity of the hydrogel itself and the substantial quantity of keratinous penetrant present in the hydrogel. The stratum corneum, the outermost layer of the skin, exhibits a high impedance, which increases the contact impedance and affects the acquisition of bioelectrical signals. The presence of the penetrant effectively reduced the impact of the high impedance of the stratum corneum on the measurement. As a bioelectrode, hydrogel should not cause allergic skin redness and swelling. The PEDOT/PAA/PVA hydrogel electrodes and the commercially available ECG electrodes were attached to the skin on the inner side of the forearm for a period of four hours. Then, the electrode sheet was removed, and the condition of the skin was examined to ascertain the presence of any irritation and to determine the contact impedance of the hydrogel. The results demonstrated that the area where the commercial electrodes and hydrogel electrodes were attached exhibited no evidence of allergic reactions, redness, or swelling, with the exception of the indentation caused by adhesion. Therefore, under the conditions of this 4 h test, the hydrogel showed no observable risk of causing acute skin irritation or allergies, suggesting its potential suitability for bioelectricity acquisition (Figure S2).

The PEDOT/PAA/PVA hydrogel exhibited conductivity and sensitivity, and it did not cause skin redness or swelling or allergic reactions, rendering it suitable for use as a skin surface electrode. In a pair of commercially available ECG electrodes attached adjacent to the PEDOT/PAA/PVA hydrogel electrodes as a control group of ECG acquisition signals, synchronized processing of their acquired ECG signals of the subjects revealed that both electrodes acquired clear ECG signal images (Figures S3 and S4), with a cosine similarity of 0.9928 between the two curves. These findings suggested that PEDOT/PAA/PVA hydrogels could be used as ECG electrodes with a high degree of safety and reliability.

**Figure 7:**
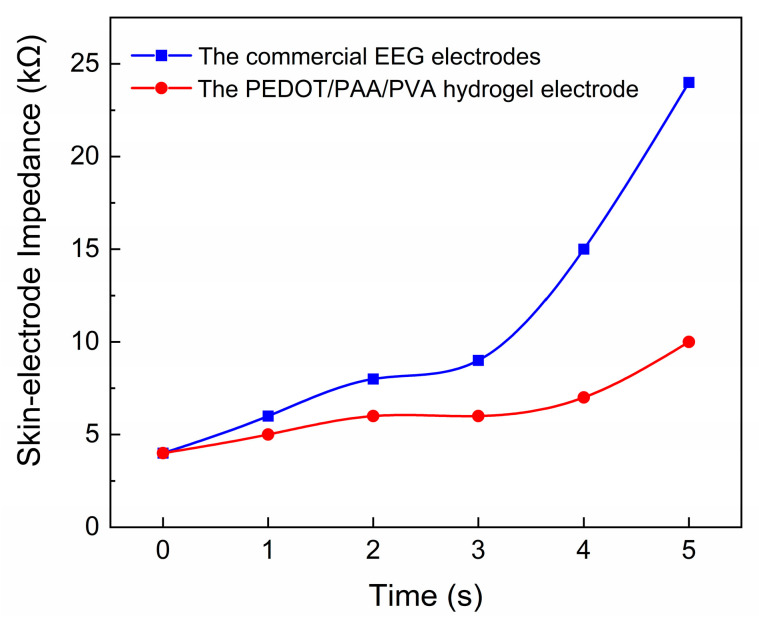
The long-term monitoring of impedance for the electrode filled with EEG paste and the PEDOT/PAA/PVA hydrogel electrode.

This approach was expected to enhance signal quality and mitigate the impact of motion artifacts on the measurements. In order to guarantee the uninterrupted procurement of EEG data over an extended timeframe, it is imperative to preserve low impedance across the EEG electrode over the course of time. The commercially available electrode was attached to the forehead in conjunction with the PEDOT/PAA/PVA hydrogel electrode, and the impedance data were continuously monitored. It has been demonstrated that the hydrogel electrode maintained a Direct Current (DC) contact impedance of less than 10 kΩ for a duration of five hours during continuous measurement (Figure 7), thereby indicating the feasibility of prolonged EEG monitoring.

**Figure 8:**
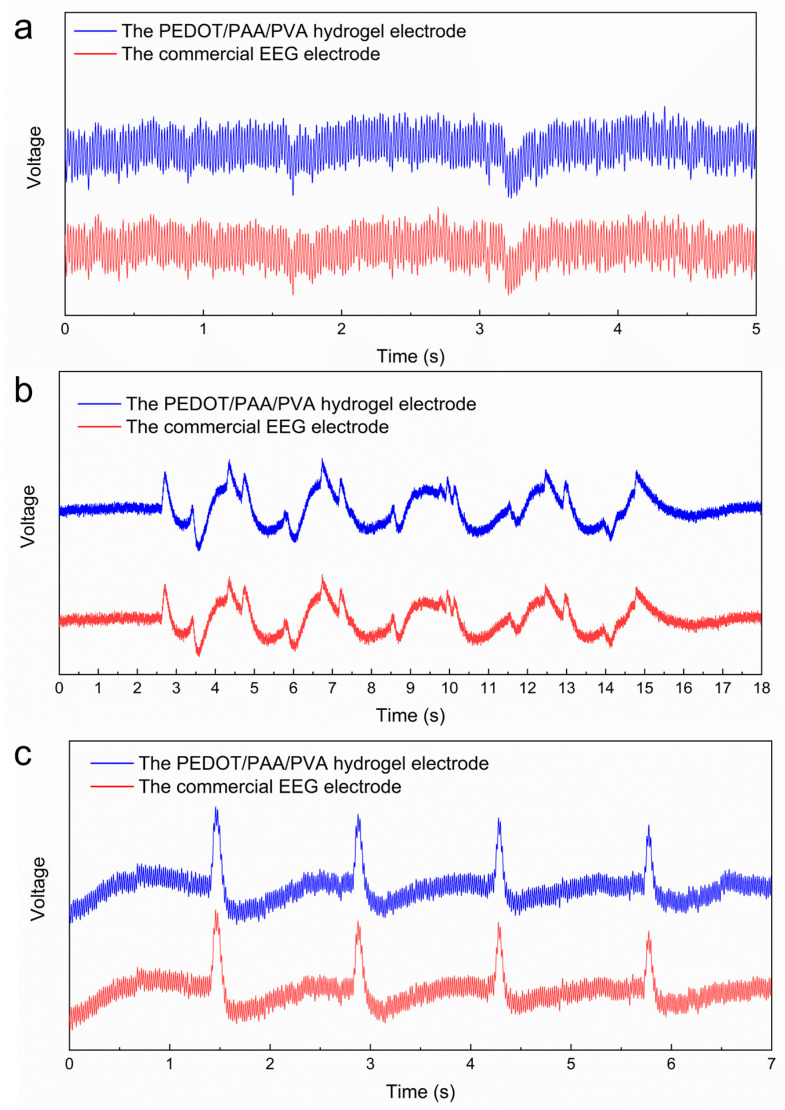
EEG signals in different states: (**a**) the signal of closing the eyes and relaxing, (**b**) the signal of blinking and (**c**) rolling the eyes.

In the context of EEG testing, a circular piece of hydrogel electrode, measuring 10 mm in diameter and 1 mm in height, was positioned at the subject’s right prefrontal test point, Fp2. Similarly, a commercially available conductive paste was instilled at the left prefrontal test point, Fp1. The EEG signals acquired by the two types of EEG electrodes were recorded simultaneously at Fp1 and Fp2. Figure 8a illustrates the EEG signals of the subject while maintaining a resting state. It can be observed that the commercial EEG electrodes and the PEDOT/PAA/PVA hydrogel electrodes yielded high-frequency signals with comparable shapes. The cosine similarity was calculated to be 0.9967, thereby substantiating the assertion that the PEDOT/PAA/PVA hydrogel possessed the capacity to measure high-frequency signals. The analysis of the collected EEG signals during regular blinking and eye rotation of the subjects revealed that the cosine similarity of the signals acquired by hydrogel electrodes and commercial EEG electrodes was 0.9970 and 0.9994, respectively (Figure 8b,c). These findings indicate that hydrogel can be used as an EEG electrode without causing skin irritation and that it possesses a reliable ability to acquire bioelectric signals.

### 3.4. Universal Applicability for the Preparation of Conductive Hydrogels

Subsequent to the substitution of various hydrogel substrates and conductive polymers in the procedure, an endeavor was undertaken to fabricate conductive hydrogels with disparate compositions employing the proposed method. The black-green Polypyrrole (PPy)/PAA and dark brown PANI/PAA solutions was obtained by subjecting pyrrole or aniline into a mixture of PAA, HCl, FeCl_3_, and APS at a low-temperature environment. The resulting PPy/PAA and PANI/PAA solutions were subsequently amalgamated with the PVA solution. On the other hand, the PEDOT/PAA solution was mixed with the Polyacrylamide (PAAm) to substitute PVA. It was observed that the mixing process resulted in precipitations, and the solution adopted a light yellow hue and became transparent, indicating the integration of conductive polymers in precipitates. The precipitates resulting from the aforementioned mixing were collected, subjected to centrifugation, dried, and shaped to yield PPy/PAA/PVA, PANI/PAA/PVA, and PEDOT/PAA/PAAm hydrogels. There was a little difference in preparing the polydopamine composite hydrogel. The PVA solution was prepared as an alkaline solution and combined with dopamine to form a Polydopamine (PDA)/PVA mixture, followed by the mixing with a blend of PAA, HCl, and FeCl_3_. The resultant mixture produced a black-brown precipitate. This precipitate was then subjected to the same procedures as other formulars. All these hydrogels exhibited favorable mechanical properties. Additionally, PANI/PAA/PVA was selected as an example to function as an ECG electrode using the same method used for PEDOT/PAA/PVA (Figure S5). The cosine similarity of the two signal curves obtained from the PANI/PAA/PVA hyrogel electrode and the commercial ECG electrode reached 0.9934, thereby demonstrating that the excellent electrical properties of the PANI/PAA/PVA hydrogel are sufficient for use as ECG electrodes. The experimental findings demonstrated the efficacy of the precipitation method in replacing both the hydrogel network and the conductive polymer in the fabrication of conductive polymer hydrogel composites. This approach not only provided new possibilities for customizing hydrogel properties but also significantly expanded the application scenarios of the precipitation method.

## 4. Conclusions

In conclusion, a novel method for the facile preparation of conductive polymer composite hydrogels was developed through a straightforward solution blending and centrifugation process. The PEDOT/PAA/PVA hydrogels exhibited excellent electrical and mechanical properties with a conductivity of 4.065 S/m. The hydrogel strain sensor exhibited a response speed of 400 ms, a sensitivity of 1.86, and good stability and repeatability. Furthermore, in a preliminary skin irritation test, the hydrogel demonstrated no signs of skin irritation under the tested conditions, and exhibited a skin contact impedance that was much smaller than that of commercial ECG electrodes. The ECG and EEG signals acquired by hydrogel electrodes and commercial electrodes showed a very high degree of similarity, thus proving the reliability of PEDOT/PAA/PVA hydrogels as bioelectrodes. Furthermore, the replacement of the hydrogel substrate and conductive polymer materials, respectively, was also successfully achieved. Consequently, this study establishes a versatile precipitation strategy for the rapid synthesis of high-performance conductive hydrogels, thereby overcoming the long-standing limitations of complex and time-consuming fabrication methods and providing a scalable platform for on-demand conductive hydrogel design.

## Figures and Tables

**Table 1:** Experimental formulation utilizing PEDOT concentration control.

Sample	EDOT	10% APS
PEDOT5/PAA/PVA	5 μL	150 μL
PEDOT10/PAA/PVA	10 μL	300 μL
PEDOT15/PAA/PVA	15 μL	450 μL
PEDOT20/PAA/PVA	20 μL	600 μL

## Data Availability

The data presented in this study are available in the article.

## Supplementary Materials

The following supporting information can be downloaded at: https://www.mdpi.com/article/10.3390/s25196032/s1, Figure S1: Continuous cyclic compression load-unloading curves of PEDOT/PAA/PVA hydrogels at different strains (20–100%); Figure S2: Skin irritation test: (a) test process (b) skin condition after test, the upper and lower core electrodes are commercial core electrodes and hydrogel electrocardiograms; Figure S3: ECG signal comparison test; Figure S4: Comparison of contact impedance between hydrogel and commercial ECG electrodes; Figure S5: Comparison of ECG test signals of PANI/PAA/PVA hydrogel and commercial ECG electrode.
