# Supplementary material for: Simple Preparation of Conductive Hydrogels Based on Precipitation Method for Flexible Wearable Devices
Source: Sensors (Basel). 2025 Oct 1;25(19):6032. doi: 10.3390/s25196032 (PMC12526617; doi:10.3390/s25196032)
Supplement: Supplementary file 1 [file sensors-25-06032-s001.zip › sensors-3857068-supplementary.pdf]

Electronic Supplementary Information for

Simple preparation of conductive hydrogels based on precipitation method for flexible wearable devices

Bolan Wu 1, Jiahao Liu 1, Zunhui Zhao 1, Na Li 1, Bo Liu 1, and Hangyu Zhang 1,*

[1] School of Biomedical Engineering, Liaoning Key Lab of Integrated Circuit and Biomedical Electronic System, Faculty of Medicine, Dalian University of Technology, Dalian, 116024, China; 2251740842@mail.dlut.edu.cn (B.W.); zhaozh_dut@163.com (Z.Z.); ljh2356486741@163.com (J.L.); lina316@dlut.edu.cn (N.L.); lbo@dlut.edu.cn (B.L.).

*Corresponding author: E-mail: hangyuz@dlut.edu.cn

This supplementary file includes:

Figure S1 to S5

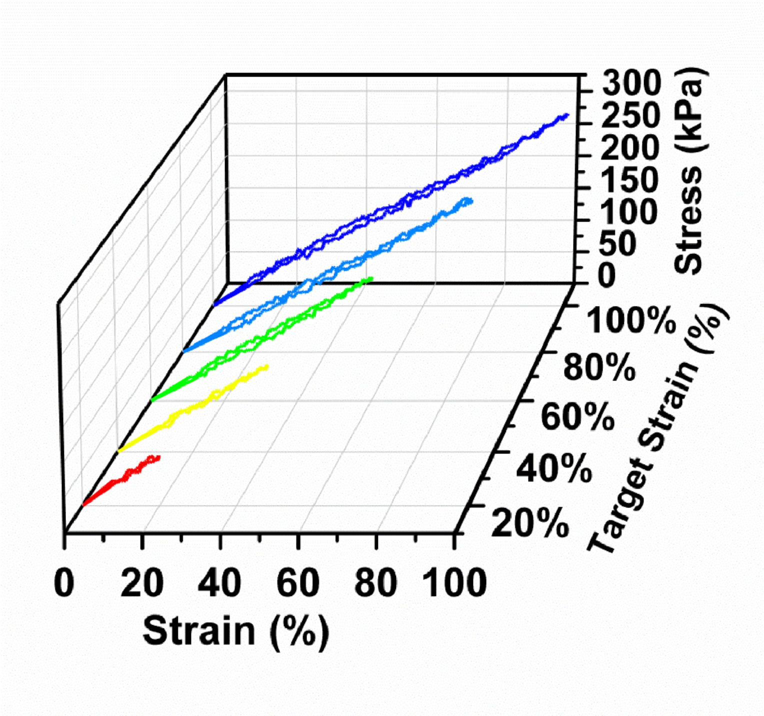

Figure S1. The continuous cyclic compression load-unloading curves of PEDOT/PAA/PVA hydrogels at varying strains (20-100%).

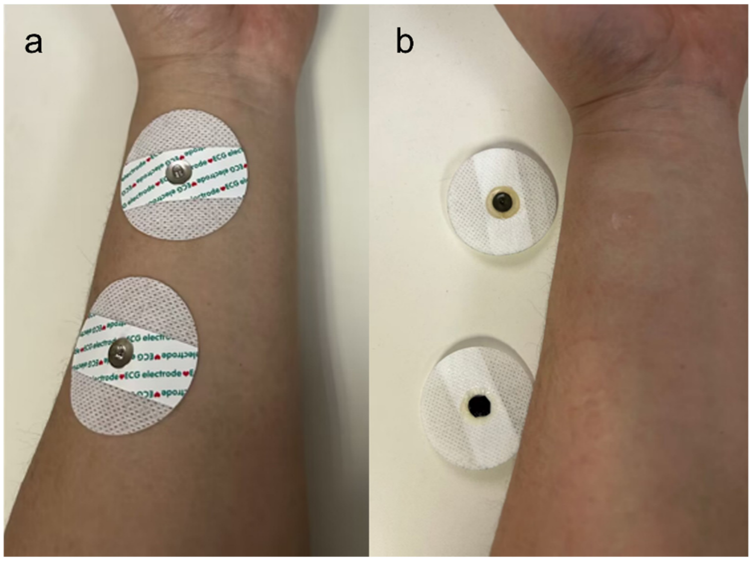

Figure S2. The effects of a particular substance on the skin. The test process(a) and skin condition after test(b). The upper core electrodes is commercial core electrode and the lower is the PEDOT/PAA/PVA hydrogel electrode.

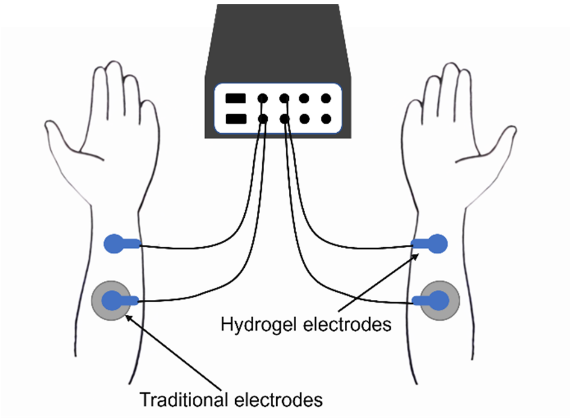

Figure S3. ECG signal comparison test

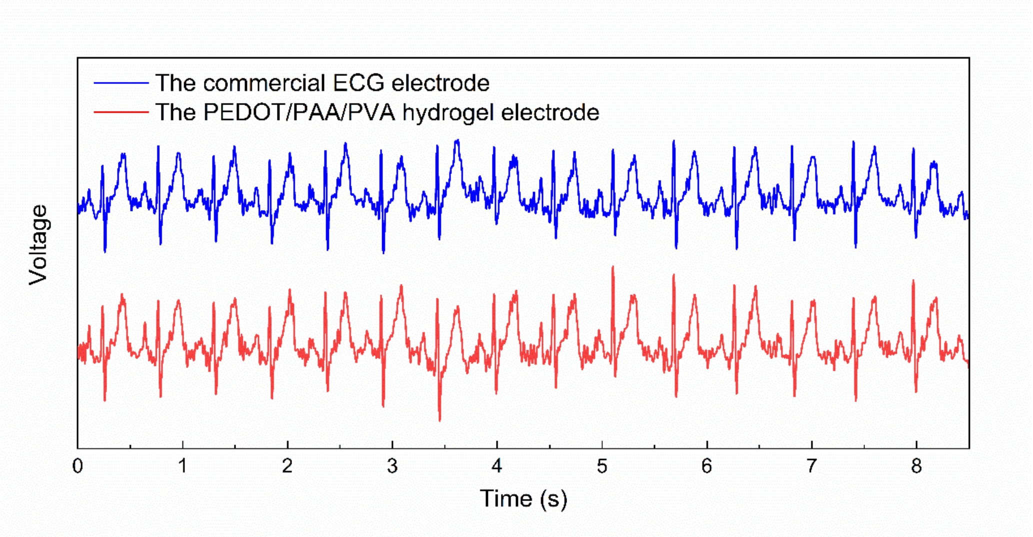

Figure S4. Comparison of the contact impedance of the PEDOT/PAA/PVA hydrogel electrode with that of commercial ECG electrodes.

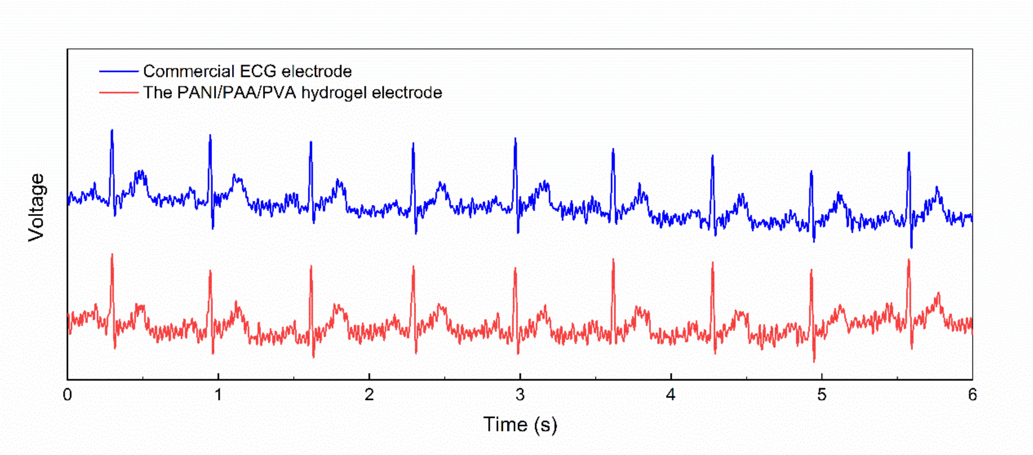

Figure S5. Comparison of the contact impedance of the PANI/PAA/PVA hydrogel electrode with that of commercial ECG electrodes.
